# Polytetrafluoroethylene fume–induced pulmonary edema: a case report and review of the literature

**DOI:** 10.1186/s13256-015-0593-9

**Published:** 2015-05-14

**Authors:** Rikuta Hamaya, Yuko Ono, Yasuyuki Chida, Ryota Inokuchi, Ken Kikuchi, Tadanobu Tameda, Choichiro Tase, Kazuaki Shinohara

**Affiliations:** 1 Department of Anesthesiology and Critical Care Medicine, Ohta General Hospital Foundation, Ohta Nishinouchi Hospital, 2-5-20 Nishinouchi, Koriyama, Fukushima, 963-8558 Japan; 2 Emergency and Critical Care Medical Center, Fukushima Medical University Hospital, 1 Hikarigaoka, Fukushima, 960-1295 Japan; 3 Department of Emergency and Critical Care Medicine, The University of Tokyo Hospital, 7-3-1 Hongo, Bunkyo-ku, Tokyo, 113-8655 Japan; 4 Department of General and Emergency Medicine, JR General Hospital, Yoyogi, Shibuya-ku, Tokyo, 151-8528 Japan; 5 Department of Radiology, Fukushima Medical University Hospital, 1 Hikarigaoka, Fukushima, 960-1295 Japan; 6 Department of Radiology, Ohta General Hospital Foundation, Ohta Nishinouchi Hospital, 2-5-20 Nishinouchi, Koriyama, Fukushima, 963-8558 Japan

**Keywords:** Acute respiratory distress syndrome, Neutrophil elastase inhibitor, Peripheral area sparing, Pulmonary inflammation, Radiological features, Teflon®, Toxic fumes

## Abstract

**Introduction:**

Polytetrafluoroethylene is ubiquitous in materials commonly used in cooking and industrial applications. Overheated polytetrafluoroethylene can generate toxic fumes, inducing acute pulmonary edema in some cases. However, neither the etiology nor the radiological features of this condition have been determined. For clarification, we report an illustrative case, together with the first comprehensive literature review.

**Case presentation:**

A previously healthy 35-year-old Japanese man who developed severe dyspnea presented to our hospital. He had left a polytetrafluoroethylene-coated pan on a gas-burning stove for 10 hours while unconscious. Upon admission, he was in severe respiratory distress. A chest computed tomographic scan showed massive bilateral patchy consolidations with ground-glass opacities and peripheral area sparing. A diagnosis of polytetrafluoroethylene fume–induced pulmonary edema was made. He was treated with non-invasive positive pressure ventilation and a neutrophil elastase inhibitor, which dramatically alleviated his symptoms and improved his oxygenation. He was discharged without sequelae on hospital day 11. A literature review was performed to survey all reported cases of polytetrafluoroethylene fume–induced pulmonary edema. We searched the PubMed, Embase, Web of Science and OvidSP databases for reports posted between the inception of the databases and 30 September 2014, as well as several Japanese databases (Ichushi Web, J-STAGE, Medical Online, and CiNii). Two radiologists independently interpreted all chest computed tomographic images. Eighteen relevant cases (including the presently reported case) were found. Our search revealed that (1) systemic inflammatory response syndrome was frequently accompanied by pulmonary edema, and (2) common computed tomography findings were bilateral ground-glass opacities, patchy consolidation and peripheral area sparing. Pathophysiological and radiological features were consistent with the exudative phase of acute respiratory distress syndrome. However, the contrast between the lesion and the spared peripheral area was striking and was distinguishable from the common radiological features of acute respiratory distress syndrome.

**Conclusion:**

The essential etiology of polytetrafluoroethylene fume–induced pulmonary edema seems to be increased pulmonary vascular permeability caused by an inflammatory response to the toxic fumes. The radiological findings that distinguish polytetrafluoroethylene fume–induced pulmonary edema can be bilateral ground-glass opacity or a patchy consolidation with clear sparing of the peripheral area.

## Introduction

Polytetrafluoroethylene (PTFE), or Teflon® (DuPont, Wilmington, DE, USA), is ubiquitous in materials commonly used in cooking and industrial applications owing to its thermal stability and non-stick properties. However, overheated PTFE generates toxic fumes that can occasionally cause acute pulmonary edema [1-16]. To date, neither the etiology nor the radiological features of PTFE fume–induced pulmonary edema has been determined [1-16]. We therefore report an illustrative case and have conducted the first comprehensive literature review to clarify the etiology and radiological features of PTFE fume–induced pulmonary edema.

## Case presentation

**Figure 1:**
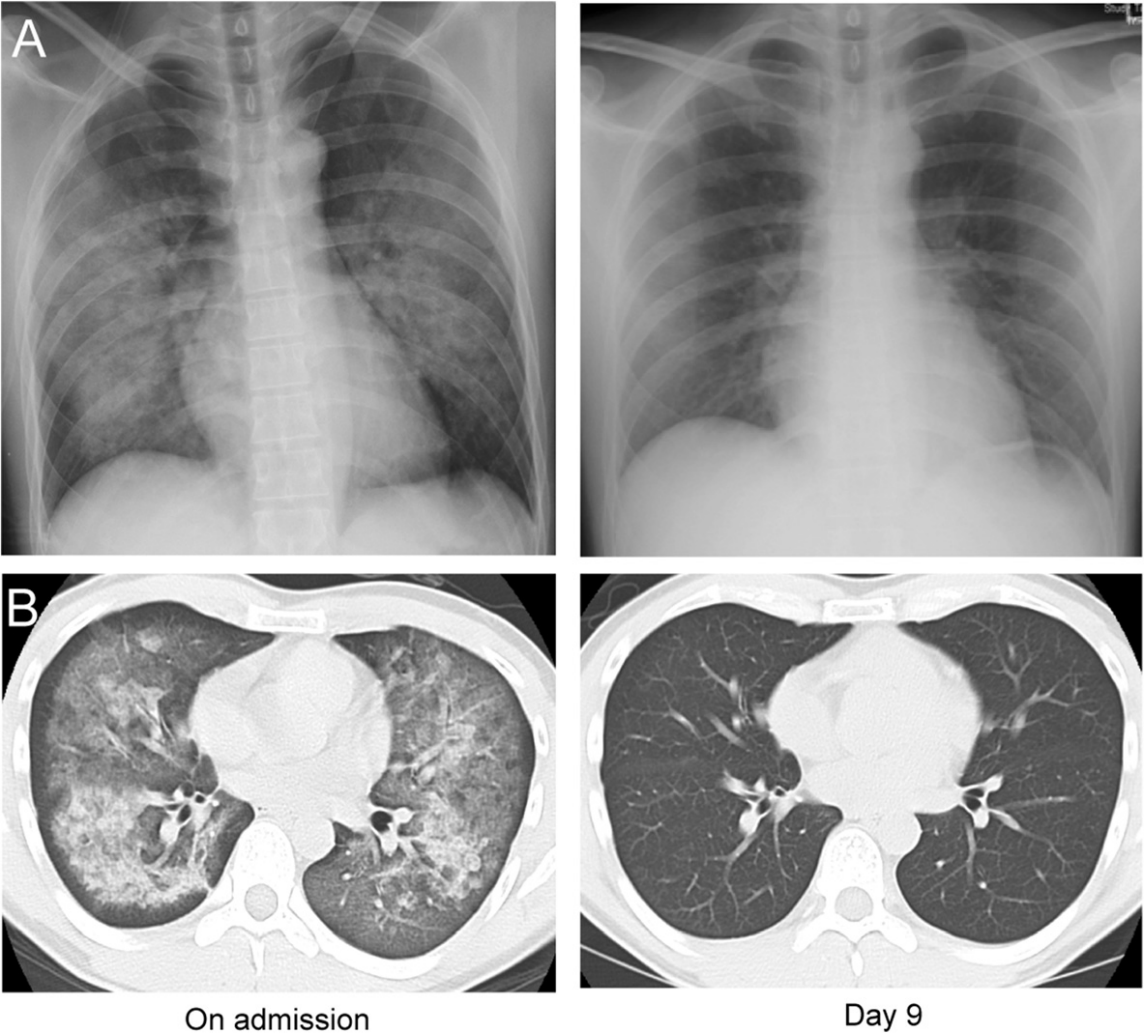
Chest X-ray and computed tomographic scan obtained upon admission and on day 9 of hospitalization. **(A)** Bilateral infiltration shadows were detected on admission (left), which had completely disappeared at day 9 (right). **(B)** On admission, bilateral patchy consolidations with ground-glass opacities and sparing of peripheral areas were found (left). On day 9 of the patient’s hospitalization, these shadows had completely disappeared (right).

A previously healthy 35-year-old Japanese man was admitted to our hospital with dyspnea and dry cough. He had fallen asleep while leaving a PTFE-coated pan on the stove, which caught fire. He awoke 10 hours later with severe dyspnea and noticed that the room was filled with white smoke. The PTFE coating of the pan was completely burned off, although the fire had not spread outside the pan. Upon admission, his vital signs were as follows: body temperature, 37.1°C; heart rate, 100 beats/min; blood pressure, 131/97mmHg; respiratory rate, 30 breaths/min; and percutaneous oxygen saturation, 98% (on oxygen 10L/min via a non-rebreather mask). The patient was alert and denied using any medications, including illicit drugs. Auscultation revealed bilateral coarse crackles. His white blood cell count was 22,100/μl with 91.2% neutrophils, and his arterial oxygen pressure was 233.5mmHg while he was on 10L/min oxygen. A chest X-ray showed bilateral infiltration (Figure 1A). Chest computed tomography (CT) revealed massive, bilateral, patchy consolidations with ground-glass opacities and sparing of the peripheral areas (Figure 1B). These lesions were distributed in a dorsally dominant manner (Figure 1B). The patient’s echocardiogram and electrocardiogram were normal, so a diagnosis of PTFE fume–induced, non-cardiogenic pulmonary edema with systemic inflammatory response syndrome (SIRS) was made. The patient was admitted and treated with non-invasive positive pressure ventilation (NPPV) and intravenous sivelestat (Elaspol®; Ono Pharmaceutical, Osaka, Japan). NPPV was initiated in a setting of positive end-expiratory pressure of 8cmH_2_O and intravenous sivelestat at a dosage of 4.8 mg/kg/day, which dramatically alleviated his symptoms and improved his oxygenation on the day of admission. His respiratory status rapidly improved, and a second chest CT scan on day 9 revealed complete resolution of the infiltrates (Figure 1B). The patient was discharged to home without any sequelae on hospital day 11.

On 30 September 2014, we searched for all reported cases of PTFE fume–induced pulmonary edema on the PubMed, Embase, Web of Science, OvidSP and several Japanese databases (Ichushi Web, J-STAGE, Medical Online and CiNii), without language restriction and using the following keywords: “polymer fume fever,” “Teflon®,” “polytetrafluoroethylene,” “pulmonary/lung edema” and “acute lung injury/acute respiratory distress syndrome (ARDS).” Three of the authors (RH, YO and RI) performed independent screenings. Cross-referencing was performed, and all the relevant case reports and studies were included. We excluded the following: (1) cases without evidence of pulmonary edema, (2) cases without an association with PTFE fumes and (3) academy meeting abstracts. The search produced 121 articles, of which 17 were potential candidates [1-16]. Next, clinical features including patient characteristics, the situation under which exposure occurred, symptoms, treatment and outcome were reviewed by three intensivists (RH, YO, and RI). One report was excluded because of insufficient information [3], leaving 16 reports and 17 relevant cases [1,2,4-16] for inclusion in this review. The temperature of the overheated PTFE was estimated based on information in the relevant reports (molding settings [4,5,11], cigarettes [17] and an overheated pan [18]). SIRS was defined according to the criteria originally proposed by the American College of Chest Physicians/Society of Critical Care Medicine Consensus Conference [19]. All CT images of PTFE fume–induced pulmonary edema were interpreted independently by two chest radiologists (KK and TT). The distribution of the disease and the dominant lesion were also noted. Thirteen reports without CT findings were excluded [1,4-14], resulting in four reports [2,3,15,16] and eight cases ultimately being eligible for inclusion in this review.

The clinical characteristics of PTFE fume–induced pulmonary edema described in this review, including our patient, are summarized in Table 1. The patient demographics of the cases in the literature review consisted of 16 men and 2 women, aged 21 to 59 years. Many patients were smokers (12 of 18), and most did not have any comorbidities (15 of 18). Among all of the reports included here, seven cases involved exposure to PTFE-containing materials in factories or laboratories, 6 cases were of patients who had smoked PTFE-contaminated cigarettes and 5 reports described exposure to fumes from overheating PTFE-coated kitchenware in the home. Common symptoms were dyspnea (17 of 18), cough (12 of 18) and flu-like symptoms such as fever (9 of 18) and chills (6 of 18). SIRS was frequently present (10 of 18). All patients had evidence of exposure to fumes developed from overheated (391 to 875°C) PTFE. One patient was exposed to PTFE fumes for 9 hours and died 5 hours after admission despite intensive treatment that included intubation [11]. Neither NPPV nor neutrophil elastase inhibitor was used in previously reported cases. Transbronchial lung biopsy was performed in one case, which revealed marked neutrophil migration into the alveoli with edema in the alveolar septa [12]. Table 2 shows the chest CT characteristics of PTFE fume–induced pulmonary edema, including our patient. Four patients underwent chest CT on the day of admission: two on day 2 and two on day 4. Common findings were ground-glass opacities (eight of eight), peripheral area sparing (six of eight) and patchy consolidation (four of eight). With the exception of a single patient, these lesions were distributed bilaterally (seven of eight) and predominantly on the back in most cases (five of eight).

**Table 1:** **Summary of clinical characteristics of polytetrafluoroethylene fume–induced pulmonary edema** ^**a**^

**Patient**	**Authors**	**Sex**	**Age (yr)**	**Comorbid disease**	**Smoking**	**Situation**	**Overheated temperature (°C)**	**Exposure time**	**SIRS**	**Symptoms**	**Treatment**	**Outcome (treatment period)**
1	Harris *et al*. [1]	Male	38	No	N/R	Heating PTFE extruder in an oven at a laboratory	N/R	N/R	Yes	Dyspnea	Absolute rest	Discharged (1 day)
2	Lee *et al*. [11]	Male	43	No	N/R	Molding PTFE-containing materials at a factory, dayshift worker	>410 to 510	9 hr	Yes	Dyspnea, malaise	Intubation, antibiotics, inotropics	Died (5 hr)
3	Lee *et al*. [11]	Male	37	No	Yes	Foreman of patient 2, with job different from that of patient 2 (monitoring, cutting and packing)	>410 to 510	9 hr (intermittently exposed)	No	Dyspnea, fever, chest pain, chills, malaise	O_2_	Discharged (7 days)
4	Lee *et al*. [11]	Male	22	No	N/R	Colleague of patient 2, a night shift worker with job different from that of patient 2 (monitoring, cutting and packing)	>410 to 510	9 hr (intermittently exposed)	No	Dyspnea, cough, chest pain	N/R	Discharged (9 days)
5	Robbins *et al*. [4]	Male	38	No	Yes	Welding PTFE-containing materials at a factory.	>560	3 hr	Yes	Dyspnea, cough, fever, chest pain	O_2_, antibiotics	Discharged (3 days)
6	Evans *et al*. [5]	Male	49	No	Yes	Molding PTFE-containing materials at a factory	740	1 hr	N/A	Dyspnea, cough, fever, throat pain	O_2_	Discharged (2 days)
7	Haugtomt *et al*. [8]	Male	33	No	N/R	Sanding Teflon-coated surface at a factory	N/R	15 min	Yes	Dyspnea, fever, chest pain, blood sputum	Diuretics, antibiotics, dopamine, O_2_	Discharged (7 days)
8	Brubaker *et al*. [6]	Male	N/R	No	Yes	Smoking PTFE-contaminated cigarettes during commute	470 to 812	<10 min	N/A	Dyspnea, cough, chest pain, chills	N/R	Discharged (N/R)
9	Patel *et al*. [13]	Male	40	No	Yes	Smoking PTFE-contaminated cigarettes in the home	470 to 812	<10 min	Yes	Dyspnea, cough, fever, chills	Albuterol	Discharged (2 days)
10	Tanino *et al*. [12]	Female	25	No	Yes	Smoking PTFE-contaminated cigarettes in the home	470 to 812	<10 minutes	No	Dyspnea, cough, fever	Steroid	Discharged (10 days)
11	Silver *et al*. [9]	Male	21	No	Yes	Smoking PTFE-contaminated cigarettes at a factory	470 to 812	<10 min	Yes	Dyspnea, cough, chills, nausea/vomiting	Antibiotics	Discharged (1 day)
12	Strøm *et al*. [14]	Male	36	No	Yes	Smoking PTFE-contaminated cigarettes in the home	470 to 812	<10 min	Yes	Dyspnea, chills	Antibiotics, O_2_	Improved (1 day)
13	Myhre *et al*. [7]	Male	25	No	Yes	Smoking PTFE-contaminated cigarettes during commute	470 to 812	<10 min	N/A	Dyspnea, chills, cough, headache	N/R	Improved (19 hr)
14	Shimizu *et al*. [2]	Male	29	No	N/R	Overheating PTFE-coated pan on fire in the home	>391	6 hr	No	Dyspnea, cough	O_2_	Discharged (3 days)
15	Toyama *et al*. [16]	Male	59	OSAS	Yes	Overheating PTFE-coated kitchenware in oven in the home	>391	4 hr	Yes	Fever, throat pain	O_2_, diuretics	Discharged (9 days)
16	Son *et al*. [15]	Male	30	BA	Yes	Overheating PTFE-coated pan on fire in the home	>391	7 hr	No	Dyspnea, cough, fever	Observation	Discharged (6 days)
17	Zanen *et al*. [10]	Female	26	Wilms’ tumor	No	Overheating PTFE-coated kitchenware in microwave oven in the home	>391	<10 min	Yes	Dyspnea, cough, fever, chest pain	O_2_, steroid	Discharged (1 day)
18	Our patient	Male	35	No	Yes	Overheating PTFE coated pan on fire in the home	>391	10 hours	Yes	Dyspnea, cough	NPPV, sivelestat	Discharged (11 days)

^a^BA, Bronchial asthma; N/A, Not available; NPPV, Non-invasive positive pressure ventilation; N/R, Not recorded; OSAS, Obstructive sleep apnea syndrome; PTFE, Polytetrafluoroethylene; SIRS, Systemic inflammatory response syndrome.

**Table 2:** **Chest computed tomography characteristics of polytetrafluoroethylene fume–induced pulmonary edema**

		**PTFE fume–induced pulmonary edema patients, n = 8 (%)**
**Graphic pattern**		
	Patchy consolidation	4 (50)
	Ground-glass opacity	8 (100)
	Peripheral area sparing	6 (75)
	Interlobular septal thickening	2 (25)
**Distribution**		
	Bilateral	7 (87.5)
	Unilateral	1 (12.5)
**Dominant lesion**		
	Dorsal	5 (62.5)
	Ventral	1 (12.5)
	No dominant lesion	2 (25.0)

CT, Computed tomography; PTFE, Polytetrafluoroethylene.

## Discussion

To the best of our knowledge, this is the first systematic review of PTFE fume–induced pulmonary edema. Because of the ubiquity of this material, all health care providers need to be aware of the characteristics of this disease. Our search revealed that (1) the essential etiology can be inflammatory pulmonary vascular hyperpermeability, (2) the radiological features can be bilateral ground-glass opacity or a patchy consolidation with clear peripheral area sparing and (3) the duration of PTFE fume exposure is a possible aggravating factor.

First, the essential etiology of PTFE fume–induced pulmonary edema can be inflammatory pulmonary vascular hyperpermeability. Flu-like symptoms and SIRS frequently accompany exposure, which are probably associated with pulmonary inflammation as a result of the toxic fumes. In one report, authors described the transbronchial lung biopsy findings in a patient with PTFE fume–induced pulmonary edema, revealing marked neutrophil migration into the alveoli with alveolar edema [12]. In a laboratory study, remarkable neutrophil infiltration and an increased level of inflammatory cytokines were found in the pulmonary lavage of rats that had been exposed to PTFE fumes [20]. Both are consistent with the pathological findings regarding the exudative phase of ARDS. NPPV [21] and neutrophil elastase inhibitors [22] are known to work effectively in treating disease of this etiology.

Second, the radiological features of PTFE fume–induced pulmonary edema can be bilateral ground-glass opacity or patchy consolidation with clear peripheral area sparing. Bilateral ground-glass opacity and patchy consolidation are consistent with the chest CT findings regarding the exudative phase of ARDS, supporting the above-mentioned etiology. However, the contrast between the lesion and the spared peripheral area was striking, and these characteristics were clearly distinguishable from the common radiological features of ARDS. One plausible explanation for the spared area is that it is more difficult for the toxic fumes to reach the peripheral alveoli; consequently, this area escapes inflammation. The other explanation is related to the characteristics of lymph flow in the lungs. Tiny particles in PTFE fumes may be removed by the lymphatic drainage system, directly or by means of macrophage ingestion and migration [23]. The lymph proceeds in two opposite directions: centripetally in the center of the lung and centrifugally in the periphery [23,24]. Centrifugal lymph flow in the lung periphery may effectively remove PTFE particles to the pleural lymphatics rather than centripetally by means of the lymph flow to the hilum [23]. Dorsally dominant infiltration can also be shown by the characteristic of the lymph flow in the lungs. Lymphatic function is known to be poorest in dorsal lungs, resulting in poor clearance of particles [23]. The above-mentioned radiologic features can be helpful in making a diagnosis.

We also noted that a temperature of approximately 400°C may be the threshold for developing PTFE fume–induced pulmonary edema in humans. Animal studies involving rats have shown the development of lethal pulmonary edema when the rats were exposed to fumes produced by overheated PTFE at around 450°C [25], which is consistent with our findings.

Finally, the duration of PTFE fume exposure is a possible aggravating factor. Lee and colleagues proposed a dose–response relationship between PTFE fume exposure and disease severity in that the most heavily exposed worker (patient 2) died, whereas less-exposed workers (patient 3, a foreman not restricted to the PTFE room; and patient 4, a nightshift molder) recovered [11]. Our survey also supports this finding. Lesser-exposed patients, such as those whose PTFE fume exposure was related to smoking, recovered quickly, whereas more heavily exposed patients, such as our patient, required longer treatment periods. As discussed, the patient who was exposed to PTFE fumes for 9 hours died despite intubation [11]. In comparison, we successfully treated a similar patient (exposed to fumes for 10 hours) with NPPV and early administration of a neutrophil elastase inhibitor, suggesting that these are suitable treatments for cases involving pulmonary edema of this etiology [21,22].

## Conclusions

Our experience with our patient, as well as our literature review, suggest that the essential etiology of PTFE fume–induced pulmonary edema is increased pulmonary vascular permeability caused by an inflammatory response to the toxic fumes. The CT findings that distinguish PTFE fume–induced pulmonary edema can be bilateral ground-glass opacity or a patchy consolidation with clear peripheral area sparing.

## Consent

Written informed consent was obtained from the patient for publication of this case report and the accompanying images. A copy of the written consent is available for review by the Editor-in-Chief of this journal.

## Abbreviations

ARDS: Acute respiratory distress syndrome
BA: Bronchial asthma
CT: Computed tomography
N/A: Not available
NPPV: Non-invasive positive pressure ventilation
N/R: Not recorded
OSAS: Obstructive sleep apnea
PTFE: Polytetrafluoroethylene
SIRS: Systemic inflammatory response syndrome
